# The hidden repertoire of brain dynamics and dysfunction

**DOI:** 10.1162/netn_a_00107

**Published:** 2019-09-01

**Authors:** Anthony R. McIntosh, Viktor K. Jirsa

**Affiliations:** Rotman Research Institute, Baycrest, University of Toronto, Toronto, Canada; Institut de Neurosciences des Systemes, INSERM, Aix-Marseille Universite, Marseille, France

**Keywords:** Dynamical systems, Epilepsy, Cognition, Neuroimaging, Computational modeling

## Abstract

The purpose of this paper is to describe a framework for the understanding of rules that govern how neural system dynamics are coordinated to produce behavior. The framework, structured flows on manifolds (SFM), posits that neural processes are flows depicting system interactions that occur on relatively low-dimension manifolds, which constrain possible functional configurations. Although this is a general framework, we focus on the application to brain disorders. We first explain the Epileptor, a phenomenological computational model showing fast and slow dynamics, but also a hidden repertoire whose expression is similar to refractory status epilepticus. We suggest that epilepsy represents an innate brain state whose potential may be realized only under certain circumstances. Conversely, deficits from damage or disease processes, such as stroke or dementia, may reflect both the disease process per se and the adaptation of the brain. SFM uniquely captures both scenarios. Finally, we link neuromodulation effects and switches in functional network configurations to fast and slow dynamics that coordinate the expression of SFM in the context of cognition. The tools to measure and model SFM already exist, giving researchers access to the dynamics of neural processes that support the concomitant dynamics of the cognitive and behavioral processes.

## VIGNETTE

Both of us like to run, partly for fitness and partly for mental health. It’s easy and you can do it almost anytime and anywhere. The thing about running is that the rules on how to do it are fairly simple, but how you do it is quite varied. Running in the heat of the summer on a beach is different from running up a hill in the forest or trying to navigate an icy trail in the winter. The point here is that while the rules for running are always the same, you would not assume that the example of running on beach serves as an accurate characterization of all running that we might do. The analogy is meant to suggest this is the approach that we use when trying to link brain and behavior. The coordination of behavior by the brain can be understood as a reflection of general rules whose specific realization depends on the current context and initial conditions.

Stated more boldly, we often assume that the expression of behavior at a point in time is sufficient to understand how that behavior is coordinated. Experimental approaches focus on the characterization of brain signal time series and how they change with manipulation. Theoretical approaches most often focus on defining functions that generate these time series. Such approaches are valid insofar as they are able to characterize the local conditions that generate the time series. If the nervous system of study can only generate that time series, then this approach will be successful.

However, a different scenario emerges when we consider that a given realization is but one of many that the brain can generate. The brain is a complex adaptive system, showing the properties of multiscale behavior, emergence, and nonlinearity (Fingelkurts, 2004; Mitchell, 2009). If we acknowledge this, then a single realization captures only a partial picture of what is possible. Changes to the initial conditions for generation of the behavior can change the realization to the point where the time series bears little resemblance to other realizations. This would be construed as “noise” in most perspectives, but the case we wish to make here is that such variations can be considered as valid expressions of the rules under which behavior is coordinated.

This perspective can be more saliently appreciated when we consider clinical conditions and the variation in expression across persons. For instance, in the case of focal damage from stroke, two persons can show similar regional damage, yet show quite different clinical outcomes (Price & Friston, 2002). Person A may be very impaired, whereas Person B shows remarkable recovery. Person B, in our framework, is less debilitated because they have more options to realize a particular behavior than Person A. The rules that govern behavior are effectively the same for both persons, but the variation in expression is greater in Person B. The stroke impairs one particular set of realizations (i.e., a specific trajectory) abolishing the behavior in Person A, but for Person B only slightly alters the execution. The differences are often explained as resilience or brain reserve, which merely relabels the outcome rather than providing a mechanism of explanation. We propose that these mechanisms can be captured in the SFM framework (Pillai & Jirsa, 2017).

## GENERAL PERSPECTIVE

We present a framework wherein complex brain dynamics can be decomposed into probabilistic functional modes. These modes are mathematically operationalized as manifolds, along which trajectories evolve as the dynamics unfold embedded in a low-dimensional space or SFM (Huys, Perdikis, & Jirsa, 2014). The collection of functional modes available in a neural network constitutes its functional repertoire, which together instantiates a complete set of potential cognitive functions and overt behaviors.

It has been acknowledged by a number of neuroscience researchers that the brain is dynamic, but how that translates to their approach to gain understanding varies widely. At one end, some consider the brain to be a simple input-output system where a signal comes in, a cascade is triggered as the signal propagates, and the system produces an output appropriate to the input (Petersen & Fiez, 1993; Posner, Petersen, Fox, & Raichle, 1988). Other perspectives, stemming from the focus on intrinsic activity in the brain, go from a unidirectional input-output system to one where the input signal itself may be modified (Deco, Jirsa, & McIntosh, 2013; Fox et al., 2005; Raichle, 2010). One expression, which falls under general category of *predictive coding*, focuses on the time series of neural signals as manifestations of internal models that the brain generates to predict its inputs and its ultimate consequences (Friston, 2010; Rao & Ballard, 1999). There is another elaboration of this that reflects our SFM framework, which we will cover shortly.

The assumption underlying much predictive coding work is that the expression of behavior at a point in time is sufficient to understand how that behavior is coordinated. Other theoretical approaches focus on defining mathematical functions for behavioral time series, while empirical studies use machine-learning algorithms to classify the time series according to the behavior they are thought to support (e.g., perceptual categorization).

There are two challenges here. First, if we were to reverse engineer a system that produces the observed time series that reflects the behavior of interest, we would not learn how the behavior itself was coordinated. Rather we would only know what generates individual time series (e.g., the action of a specific set of brain areas). Second, and more problematic, is that the model would not be able to generate new behaviors that we had not previously measured. Said differently, we may be able to predict what the system *has done*, but cannot predict what it *will do*. One remedy is to update the model in light of the new behavior and to build a lookup table that relates the configuration of neural dynamics to a specific behavior. The process continues until at some point we have cataloged all the behaviors of the system. Although this sounds cumbersome, you see it played out in modern neuroscience. In neuroimaging, for example, we started with the characterization of activated brain regions and relating that to specific behavioral functions (vision, audition, language, memory), drawing inferences on the unobservable processes that were needed to instantiate such functions. We are now in the era of brain networks, where the coherent interactions between regions are the substrate for function (default network, salience network, dorsal attention network). A great deal of research now emphasizes the system characteristics that support these networks by looking at graph theory metrics (Bullmore & Sporns, 2009; Rubinov & Sporns, 2010) and by characterizing a feature of the dynamics, such as scale-free behavior and criticality (Beggs & Plenz, 2003; Haimovici, Tagliazucchi, Balenzuela, & Chialvo, 2013; Petermann et al., 2009; Tagliazucchi, Balenzuela, Fraiman, & Chialvo, 2012). If we pause and examine these observations, we have indeed done a good job of characterizing *what* the system does, but have no idea *how and why*.

The SFM framework takes a different approach, which still assumes that the brain constructs models of the world (as in predictive coding), but takes the focus from the specific instantiation of that model (aka the individual trajectory) to discovering the rules that the brain uses to develop these models. This is a subtle, but critical, difference. For SFM, the emphasis is specifically on these changes, where a given class is considered only one of the potential sets of behaviors that can be realized. Changing the configuration to encapsulate a new set is explicit in the SFM framework. The SFM framework emphasizes the architecture necessary to build those realizations, but also others that may not have been observed previously, but are a consequence of the architecture. Examples may be symmetry constraints imposed upon an architecture, allowing explicitly for symmetric and antisymmetric solutions, even though only symmetric solutions had been observed previously. Another illustration that makes the distinction between the emphasis on a specific realization versus a model for the rules that generate the realization comes from an example of calculating 3 times 4, 3 times 5, and then switch to 13 times 14. The majority of people will rapidly access their semantic memory for the first two cases, but evoke a different model to compute algorithmically the last. If the result of the computation is not in memory, then no solution can be found, whereas in the algorithmic case solutions for number computations may be found that have never been computed before. The most innate and pertinent characteristic of the brain is its capacity to generate dynamic models.

As we describe the option that encapsulates SFM, it is useful to borrow an analogy from J. H. Holland on the game of chess to illustrate the difference (Holland, 2014). One can learn chess by watching a game and tracking the movements of each piece, repeating the observation for subsequent games and then build a catalog of moves and counter moves. This is a formidable challenge given that, by rough calculations, there are at least 10^50^ possible legal move sequences, which is larger than the estimated number of atoms in the universe. The more efficient approach is to define the rules that determine the legal moves. By doing this for chess, we dramatically reduce the problem from an essentially infinite space to one where a dozen or so rules capture all possible realizations of the chess game. Mastery of chess is achieved when individual moves are combined and orchestrated into larger motifs, further classified into aggressive, defensive and strategic patterns. We understand chess by understanding the rules of play, and understand it deeply by using these rules to build coordination motifs. And this is the option that motivates the description of SFMs: *the goal for understanding brain and behavior is to determine the rules that govern the coordination of behavior*.

## CONCEPTUAL DESCRIPTION OF STRUCTURED FLOWS ON MANIFOLDS

The SFM framework lies firmly in the ideas of complex adaptive systems. Our exposition will thus borrow heavily from analogies of other, nonneural, systems that illustrate key principles to build our case, such as emergence, nonlinearity, motifs, flows, and internal models.

The notion of SFM formalizes some key general properties of complex adaptive systems. The use of the term *flows* in SFM emphasizes the dynamic nature of brain processes, where the flow formalizes the rules that enact the *internal model* of the system. The *nonlinearities* of the system impart other properties, such as *aggregation* and *emergence* that link the actions at one level of the system (e.g., network dynamics) to actions at another (e.g., behavior). The elements (or more often called “agents”) can operate at different *timescales*, and the interactions between scales are a critical feature in controlling the flow of the system. Fast timescales may have no overt consequence until slower moving scales reach a certain *tipping point*, or *bifurcation*, and the flow of the entire system changes.

**Figure 1.**
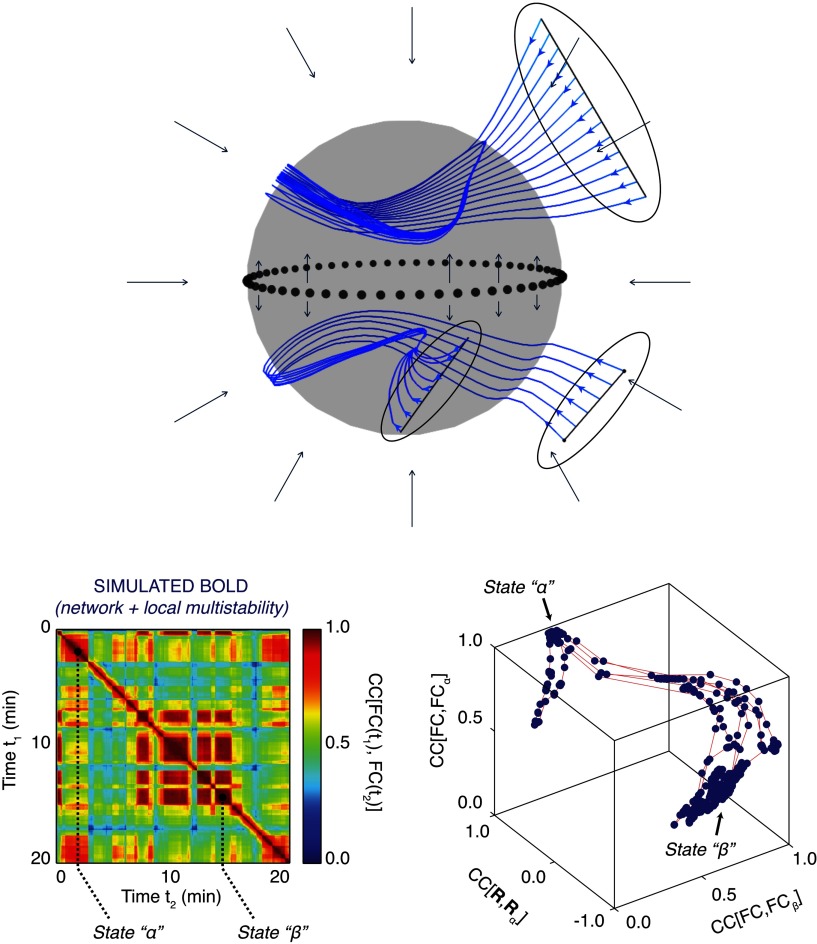
Structured flows on manifolds (SFMs). Upper figure shows a spherical attractive manifold, displaying various sets of initial conditions (black ovals) of trajectories (blue), evolving rapidly toward the manifold and then continue evolving on the manifold on a slower timescale. The timescale separation is evidenced by the large angle between trajectory and manifold (around 90 degrees). The flow on the manifold is split into two domains, one lower and one upper hemisphere, partitioned by a seperatrix (dotted line). The trajectories trace out lines on the manifold, following the flow (black arrows). The SFMs display a bi-stable organization with closed circular orbits on both hemispheres. The two lower figures show a similar organization, as captured by BOLD signals simulated with TheVirtualBrain (Hansen, Battaglia, Spiegler, Deco, & Jirsa, 2015; Sanz Leon et al., 2013). On the left, functional connectivity dynamics (FCD) are shown over 20 min, in which two large segments of invariant functional connectivity (FC) are identified as states alpha and beta. For both time windows, a principal component analysis was performed spanning state-characteristic subspaces by the leading principal components. When the BOLD signals were projected into the characteristic subspaces, the trajectory of the brain signal is unfolded, identifying the manifolds and trajectories of the corresponding states (figure on bottom right).

SFM approaches emphasize the manifolds that can be understood as force fields generating the ensemble of all possible trajectories (or flows), and are thus a mathematical expression of the rules underlying the generation of behavior. Figure 1 demonstrates the general idea of flows on manifolds with one toy example of a spherical manifold having two attractor states or domains that support different flows. Depending on the initial condition of a given trajectory, a flow evolves rapidly to the manifold and then continues on the manifold at a slower timescale. The figure also demonstrates a comparable manifold architecture in simulated resting-state functional MRI data, where changes in functional connectivity dynamics (FCD) switch two states that span a manifold.

The link of SFM to flows and emergence can be conceptualized by considering a piece of music. The analogy of the “brain as a symphony” has been made often, and is used to illustrate the fact that the emergence of function comes not from the action of a single brain area, but rather the coordination among all elements (unlike a symphony, however, in the brain there is no conductor). SFM theory provides a formal framework for these concepts of “brain as a symphony.” In a symphony, one can isolate the individual instruments to characterize their unique contribution, but it is difficult to appreciate its role in the symphony without considering the relation to other instruments. The statement “The whole is greater than the sum of its parts” is appropriate here for both the symphony and the brain.

We can further develop this analogy to build the intuition about SFM, particularly in the context of how different temporal flows (e.g., melody and piano lines in a simple song) interact in supporting the emergent behavior (the whole song). The melody and harmony often move in *different timescales*. Each can be comprehended on their own, but in a well-composed song, the relation between the lines brings a richness that is not present in either alone (e.g., *aggregate property*). This fluctuation between the melody and harmony evolves throughout the song. It is common in classical pieces for the opening melody to be repeated as a motif, but over a slightly different piano line, which may completely change the mood of the piece.

In the brain, a parallel to the symphony analogy can be drawn. As the instruments in the orchestra and musical abilities of the artists define constraints upon the symphony emerge, the anatomical connectivity and dynamic characteristic of the brain regions (network nodes) specify the rules for the evolution of dynamics. As we shall see below, this is far from a trivial constraint, as the anatomy helps define any spatial and temporal constraints for potential network configurations. For example, all things being equal, it is more likely that adjacent regions in occipital cortex will interact rather than occipital and frontal regions, simply because the occipital and frontal areas have few connections between them, and those that are connected indirectly at a long distance, imposing a longer time delay for transmission. Thus, anatomy establishes a deterministic architecture that prevents random manifolds and flows from occurring. This architecture, set atop the (nonlinear) dynamics of neurons and connected populations of neurons establishes the set of *motifs* that are available for the brain to combine in the coordination of behavior (Sporns & Kotter, 2004). Motifs in the brain have been identified from an anatomical and functional perspective (Mohajerani et al., 2013; Sporns & Kotter, 2004), and may also be related to the collection of so-called “resting-state” networks that arise through intrinsic activity (Damoiseaux et al., 2006; Fox et al., 2005). The SFM framework makes use of these motifs to articulate the functional possibilities as different motifs are realized. For example, the functional connectivity patterns in Figure 1 show that the constituents of the resting-state networks can recombine across time, forming different networks as the entire system moves across a trajectory. Thus, the motifs can be recombined to enact a variety of functional outcomes. We can refer to these as *functional* modes to emphasize that they can be both actual and potential configurations.

The asymmetries in the brain’s space-time structure, set by the structural connectivity, establish a potential for multiscale actions (Deco et al., 2013). The multiscale temporal character of these modes is founded on the fact that complex processes arise in an organism-environment context that inherently covers multiple scales. Armed with functional modes as essential building blocks, we propose additional dynamics (called operational signals) on timescales slower and faster than that of the modes. The slower process effectively binds functional modes together into sequences. More precisely, the given functional mode emerges via a competition process to temporally dominate the functional dynamics, after which it destabilizes and gives way to another mode (Haken, 2006; Perdikis, Huys, & Jirsa, 2011). The transient dynamics between modes can be triggered either by “internal” events (as in preconstructed sequences) or by “external” ones (such as perceptual events). Once engaged, the temporal attractivity of a mode guarantees functional robustness, whereas transitions between modes underlie flexibility for meaningful changes. Further variability in the function may arise via additional dynamics operating on times scales faster than (or similar to) that of the modes. Accordingly, brain function is organized in multilevel spatial and temporal hierarchies.

The hierarchical architecture is central to effective information processing, where different temporal and spatial scales interact in moving the system through behavioral repertoires. Information provided to the brain system is meaningful if and only if it qualitatively changes the “state” that the brain occupies at that moment. If an incoming signal does not change the state, then the information was not meaningful, and the incoming signal could equally have not been present. Thus, although local dynamics may change dramatically, they may not have an appreciable effect on the trajectory of the network, and thus rather than change the flow to a new part of the manifold, may only result in a trivial variation in the current trajectory. If these local dynamics intersect with larger scale dynamics at a critical point, this can establish a new trajectory for the system, either within an existing SFM or moving to a new SFM and hence a new emergent behavior. The heterogeneity of such effects has been explored using TheVirtualBrain, where Spiegler et al. (Spiegler, Hansen, Bernard, McIntosh, & Jirsa, 2016) demonstrated that the dissipation from focal stimulation across cortical and thalamic site was not uniform, where some stimulation effects did not propagate beyond the local areas while others engaged broad networks that could be related to resting-state networks. Complementary work from Deco et al. (2013) has shown that during spontaneous activity, certain nodes can act to “ignite” the reformation of functional networks, which would be consistent with a qualitative change in state. This is not to say, however, that signals that result in state changes are somehow more “conscious” than other. Indeed, many network operations will engender state changes, but not be accessible to consciousness, such as brainstem autonomic functions. Conversely, there may be behavioral changes that arise from unconscious processes, such as implicit learning, that by necessity would need to also involve state changes in brain dynamics. In this sense, the general notion of an SFM is essentially agnostic to the overt awareness of the behavior processes it supports, but does lead to the interesting speculation that there may indeed be a meaningful difference in the configurations of SFM that are consciously accessible versus those that are not.

## MATHEMATICAL DESCRIPTION OF SFM

Some of the mathematical details that define SFM have been described fully elsewhere (Huys et al., 2014; Pillai & Jirsa, 2017). Here we provide essential details to enable the link to our current narrative. SFMs are the mathematical objects capturing the dynamic properties required from a system capable of the behavior we have discussed thus far. The system under consideration is high-dimensional with *N* degrees of freedom and highly nonlinear. In order to allow for this system to generate low-dimensional behavior, that is, *M* dimensions with *M* < < *N*, there must be a mechanism in place, capable of directing trajectories in the high-dimensional space toward the *M*-dimensional subspace. Mathematically this translates into two components that are associated with different timescales: first, the low-dimensional attractor space contains a manifold *f*(.) and attracts all trajectories on a *fast* timescale; second, on the manifold a structured flow *g*(.) prescribes the dynamics on a *slow* timescale, where here slow is meant in comparison to the fast dynamics toward the attractor. For compactness and clarity, imagine the state of the system is described by the *N*–dimensional state vector *q*(*t*) at any given moment in time *t*. Then we split the full set of state variables into the components *u* and *s* where the variables in *u*define the *M* task-specific variables linked to emergent behavior in a low-dimensional subspace (the functional network), and the *N* − *M* variables in *s* define the remaining recruited degrees of freedom. Naturally, *N* is much greater than *M*. If the manifold *f*(.) is smooth and differentiable, then constraints can be established to guarantee local stability and all the dynamics is attracted thereto (Pillai & Jirsa, 2017). The manifold is given by *f*(.) = 0 and all points on the manifold are stationary points for = 0. If this is sufficiently small, then a flow emerges within the manifold, which is approximately independent (again via timescale separation) from the shape of the manifold. When this increases, then this independence is no longer a good approximation.

$$\begin{array}{c} {\dot{u}}_{j}=-f(u_{j},s_{i})u_{j}+\mu g(u_{j},s_{i})\\ {\dot{s}}_{i}=-s_{i}+N(s_{i},u_{j})\\ u\in {ℜ}^{M},s\in {ℜ}^{N-M},N>>M \end{array}$$

The flow of the nonlinear dynamic system is the right-hand side of the differential equations. In state space, the flow is a form of force field that drives the state of the system along a trajectory. The tracing out of the trajectory is the evolution of the complex dynamic system, and the flow is the rules that underlie the behavior. The above mathematical representation via a timescale decomposition is not unique, and there may be other equivalent representations capable of capturing the same flow in state space. However, the current representation is attractive for two reasons: 1) it provides a clear separation of the timescale via the smallness parameter *μ*, where the slow timescale is *μ* < < 1 and the fast timescale is on the order of 1; and 2) the current form has been successfully linked to networks composed of neural masses, coupled via multiplicative coupling functions, which are fundamental for the emergence of SFM (Pillai & Jirsa, 2017; Woodman & Jirsa, 2013). These multiplicative properties are at the heart of conductance-based modeling as embodied by the Hodgkin-Huxley equations, as well as essential in synaptic couplings. Mathematically the multiplicative coupling enables the manifold to be described globally, rather than only locally as has been the case previously in formal theories of self-organization, such as Synergetics (Haken, 1996, 2006). The formulation of SFM is a general framework, and the link to neuroscience is accomplished, for instance, when SFMs are derived from neural network equations. In these situations, the state vector *q*(*t*) is the vector of all activation variables across all brain regions, and the SFM is the mathematical representation of the dynamics of the brain network. We will provide examples in the following of applications of SFM theory to neuroscience problems, which will in all cases refer to the state vector as neural activations. It is nontrivial and not lost on us that the emergent SFM in brain activation space does not necessarily map isomorphically onto the low-dimensional dynamics (and thus SFM) in behavior. In other words, the lawfulness and rules underlying cognitive architectures may not be isomorphically related to the rules governing its directly associated brain dynamics. As attractive as such isomorphism conceptually may be, it needs to be demonstrated empirically.

## MODELING SFM IN EPILEPSY

A consideration about pathologies in the brain adds a critical element to our reflections on SFM and model emergence in the brain. Although the establishment of the SFM framework preceded the work on epilepsy, fundamental modeling of epilepsy has led to the postulate of the existence of a slow variable that dictates the expression of faster seizure activity (Jirsa, Stacey, Quilichini, Ivanov, & Bernard, 2014). During epileptic seizures, the firing activity of billions of neurons becomes organized so that oscillatory activity emerges that can be observed in electrographic recordings. This organization greatly reduces the degrees of freedom necessary to describe the observed activity, from single neurons firing to a few oscillatory collective variables. On the other hand, these oscillations trigger a series of processes at the microscopic level that slowly leads toward the end of the seizure. These slow processes can also be described by a collective variable, the permittivity variable that represents the balance (or imbalance) between the slowly varying pro- and antiseizure mechanisms. *The fast variables span an SFM and the slow variable guides the brain system through the creation and annihilation of the SFM*. The composition of fast and slow variables in epilepsy is called the Epileptor.

**Figure 2.**
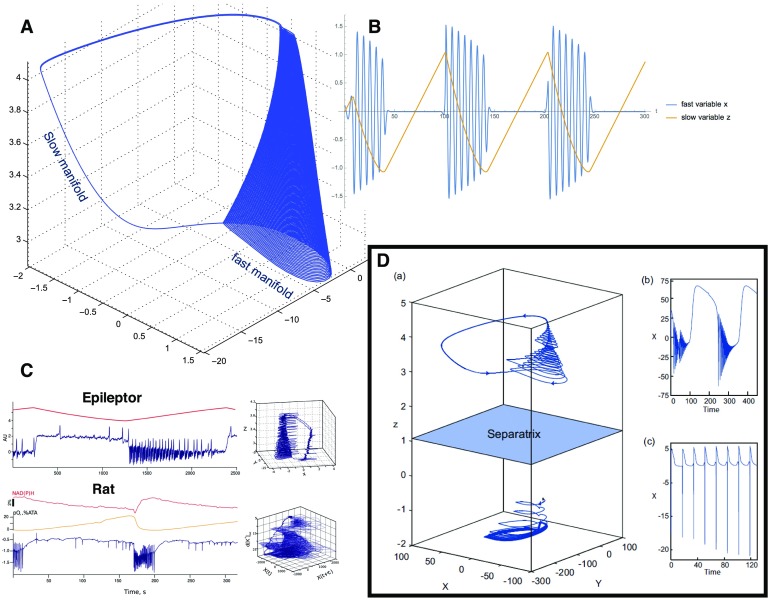
SFMs in Epilepsy. (A) Ictal and nonictal discharges have been characterized in nonlinear dynamics by two manifolds, a slow one-dimensional manifold illustrating the nonictal resting state and a fast oscillation tracing out trajectories on a cone. (B) The corresponding time series are shown on the top right, whereas the canonical SFMs are shown in panel A. (C) Two situations (time series, SFM) are shown for a detailed model signal (top trace: Epileptor) and an empirical signal (rat, in tuto hippocampus), showing the identical topological features in state space. (D) In the box on the bottom right, a state space is shown in (a), in which the upper region holds an Epileptor attractor and the lower region, separated by a separatrix (indicated in light blue), a so far unknown attractor, which is hypothesized to be linked to refractory status epilepticus. The respective time series from the two attractor spaces are plotted in the two right subpanels (b) and (c).

Biophysical parameters that slowly change in the period preceding a seizure and during the ictal state are, for example, extracellular levels of ions (Heinemann, Konnerth, Pumain, & Wadman, 1986), oxygen (Suh, Ma, Zhao, Sharif, & Schwartz, 2006), and metabolism (Zhao et al., 2011). We can thus describe the evolution of a seizure with a few collective variables acting on different timescales: fast variables that, depending on the value of their parameter, can produce either resting or oscillatory activity with bifurcations separating the different regimes; and slow variables describing the processes that brings the fast variables across the onset and offset bifurcations (Figure 2).

Across multiple patients (Jirsa et al., 2014), most had seizures characterized by different bifurcations in different moments, which implies that different classes of seizure types coexist and can be described with the same model, so that ultraslow changes in the parameters of the fast variables can bring the patient closer to one or the other seizure type. From the perspective of dynamical system modeling, this states that there must exist some slow variable dynamics (under the assumption of autonomous systems). If the slow variable exists in pathological conditions, we make the assertion that slow variable dynamics plays an equally important role in healthy conditions evolving together with the fast variable dynamics as the actual emergent subsystem, or in Haken’s words “order parameters.” The novelty here is that the emergent order parameters have an intrinsic timescale separation and comprise fast and slow variables, and not the typically single timescale of Synergetics. Fast variables act on slow variables and vice versa. The mutual presence of multiple timescales in the emergent system, the SFM, is reflective of the adaptive nature of the brain.

## HIDDEN CAPACITY OF NETWORKS REVEALED IN THE SFM FRAMEWORK

We noted earlier that a distinct advantage of creating models of rules governing coordination of behavior is the possibility of identifying novel configurations that had not yet been expressed or observed. This advantage can be illustrated from further elaboration of the Epileptor model. An exploration the dynamics across parameter ranges of the model provides confirmation of the interplay of fast and slow variables in moving the system from a quiescent phase, into seizure, and then back out. A broader parameter search identified another SFM, in which the system engaged in broad slow oscillations (Figure 2, bottom right) (El Houssaini, Ivanov, Bernard, & Jirsa, 2015). Phenomenologically, these trajectories resembled what is seen in refractory status epilepticus (RSE). The critical aspect of this observation was that this repertoire was not obvious in the initial creation of the model, but this “new behavior” was in fact part of the lawful behavior of the system.

The second important aspect of this was the observed dependencies of the seizure and RSE behaviors, wherein modification of slow variables allowed a transition between behavior, which was confirmed in animal models (El Houssaini et al., 2015). This is also a vital observation clinically as it suggests a different treatment path to alleviating RSE is to reestablish seizure rather than eliminate the dynamics all together.

By modeling the system, rather than a given realization, we were able to identify this hidden state that would be invisible to other approaches that attempt only to characterize the time series/realizations. As we noted earlier, even if one captures a large number of realizations, the quantification of these only is relevant to the particular behavior and not to the function of the system. Modeling the system, similar to what we propose in with SFM, captures both what the system does when you are watching and what it could do when you are not. The Epileptor perfectly embodies this where the model captured the presence of the RSE state, even though the system did not need to generate a realization to know that the state existed.

The Epileptor model gives a very salient demonstration of the use of the SFM framework under “disease potential.” This yields from two postulates. The first stems from the physiological fact that anyone’s brain has the potential to show seizures given the right conditions. From the SFM perspective, what this suggests is that “seizure” is an existing repertoire in anyone’s brain that can be expressed when the parameters are right (Jirsa et al., 2014). The phenomenological model provides a useful characterization of the state changes that need to occur in order to shift the flows on the manifold to the seizure attractor. A further exploration of the Epileptor model indicated that another behavior can be expressed, namely that of RSE, again once the control parameter changes are sufficient to move from the seizure attractor to the RSE attractor.

The second postulate stems from the first. If epilepsy is a part of the natural repertoire of the brain, can other clinical conditions be similarly regarded? At face value, the suggestion would be “no” because epilepsy may be an inherent biophysically property of oscillatory networks, while other scenarios arising from acquired brain injury or neurodegenerative disorders may not be equally represented across the population. But perhaps we can recast the perspective somewhat. As an adaptive system, the brain is in a constant state of testing new configurations to enhance capacity (Minerbi et al., 2009; Ziv & Brenner, 2018). Although this is generically considered plasticity, these reconfigurations seem to be spontaneous and persist when the outcome is adaptive, which is a property of complex adaptive systems. These changes are considered atop a more stable repertoire, which, usually, prevents a catastrophic situation where a maladaptive configuration is reinforced. This gives us a segue to consider maladaptive responses in terms of clinical outcome. It may be the case that brain disease expression reflects maladaptation. This would explain the observations where two persons with ostensibly the same damage can show markedly different clinical expressions, one showing severe impairment and the other showing much less, if any. In the first case, there is an attempt to adapt the damage, but the new manifold or attractor that emerged was maladaptive, resulting in a dysfunctional realization. In the second case, the adaptation was more robust, allowing the person to maintain more adaptive manifold, reducing the clinical severity. Thus, unlike the epilepsy case where seizure is a naturally part of the brain’s repertoire, in other cases the clinical expression is reflected in a given brain’s capacity to adapt to a pathological process.

These two postulates can be unified under the idea that the facility with which one moves from one manifold to another will dictate clinical outcome. For epilepsy, many persons will never have a seizure, suggesting that despite the existence of the seizure manifold, the system configuration is such that moving to this manifold never happens. In the case of perturbation from acquired brain injury or degenerative disorders, the maladaptive response comes because the existing system repertoire was not able to accommodate the perturbation. Where the clinical outcome is less severe, the perturbation still has a negative effect, but the existing repertoire is able to adapt sufficiently so as to limit disability (for a complementary perspective see Corbetta, Siegel, & Shulman, 2018).

The perspective changes the way we consider clinical progression from one where the brain is static and the clinical expression is simple loss of function to one where the clinical progression is an expression of the continual adaptation of the brain. The adaptation itself may indeed be as debilitating as the triggering event.

If this is true, then it should be possible to characterize the capacity of a given brain to adapt to negative perturbation by construction and exploration of a person’s SFM. An even more intriguing potential is that such a characterization may suggest a course of intervention that makes use of the capacity of a given person to traverse their SFM and adapt.

## FUTURE DIRECTIONS AND FINAL THOUGHTS

Our final example from the Epileptor model emphasized the interplay of fast and slow dynamics that both govern fast spiking behavior and the qualitative shift in dynamics to RSE. Saggio, Spiegler, Bernard, and Jirsa (2017) have provided a comprehensive characterization of interplay of fast and slow dynamics in and across most types of bursting behavior observed empirically beyond that which characterizes seizures. Fast and slow processes are also liable to play out in the expression of normal behavior and cognition. Obvious examples are circadian rhythms and hormonal fluctuations that modulate excitability, and even more extreme would be the relatively show maturation and aging changes that change manifold configurations, which would have the effect of enabling or eliminating flows. Neuromodulatory effects would also be candidates for slow fluctuations, here biasing the accessibility to some flows (Shine et al., 2019). The switching behavior reported for functional connectivity dynamics (e.g., Figure 1) would also presumably be linked to slower timescales. Finally, in consideration of a comparable scale interplay in the spatial domain, local and distribute spatial processes would similarly be linked to fast and slow timescales, whose expression can be related to cognitive function. Indeed, we have observed changes in scale dependency linked to cognitive performance and to maturation changes and aging (McIntosh, 2019). It is worth noting that the perspective of interacting scales does not require an appreciable change in the notions of the cognition per se, but does emphasize a more dynamical perspective where cognition is conceived in term of explicit flows rather than punctate states. To paraphrase our earlier point, the fast processes represent a trajectory on a given manifold, and the slow processes guide the brain system through the creation and annihilation of manifolds as the entire cognitive flow is elaborated.

There already exists a growing body of work that characterizes neurophysiological data by using dimensionality reduction techniques, which is a step toward defining low-dimensional manifolds that constrain network flows (Gallego et al., 2018). Indeed, recent work in functional neuroimaging is focusing on the configurations of functional networks and the changes in their configurations in relation to behavior (Khambhati, Sizemore, Betzel, & Bassett, 2018; Shine et al., 2019). Analysis of functional connectivity dynamics (Figure 1) (Hansen et al., 2015; Hutchison et al., 2013) provides a relatively straight path to manifold estimation.

**Figure 3.**
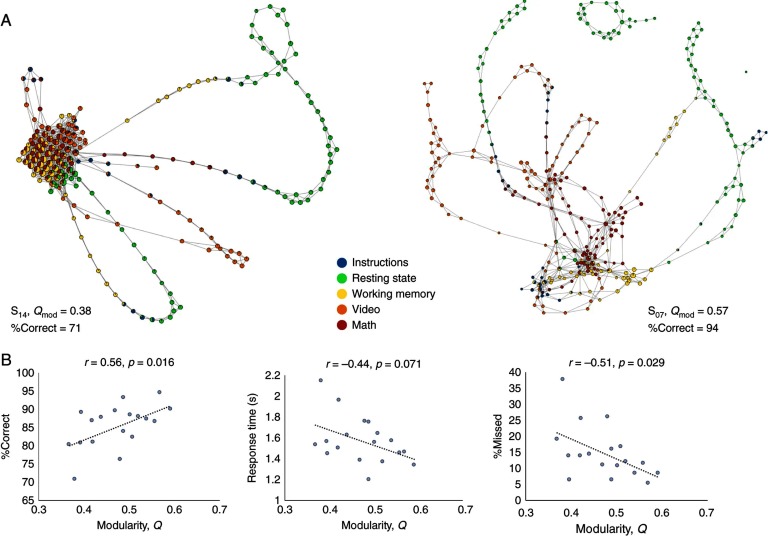
Excerpt from Saggar et al. (2018) showing the comparison of shape graphs constructed using topological data analysis of the evolution of brain activations measured with fMRI. (A) Graphs for two subjects are shown and were quantified based on modularity (Q_*mod*_) and showed a wide difference in performance (%Correct), with S_14_ (left) showing low modularity and S_07_ (right) showing higher modularity. (B) The correlation between modularity indices across all subjects and different aspect of behavior. The pattern suggests subjects with higher modularity, which may suggest a more complex manifold architecture, have better behavior.

There are established methods for manifold estimation that extend beyond functional connectivity and instead define the space (i.e., manifold) that constrains the variance of specific neurophysiological signals. Here, trial-by-trial signals are considered together to define the dimensionality of the system and then characterize the manifold features (Gallego, Perich, Miller, & Solla, 2017). Methods such as principal components analysis can give access to the manifold space (Banerjee, Tognoli, Assisi, Kelso, & Jirsa, 2008), while others explicitly characterize the manifold such as Stochastic Neighborhood Embedding and Uniform Manifold Approximation and Projection (Ma & Fu, 2011). Algebraic Topology methods are also proving to be powerful complementary techniques by giving access to geometrical characterizations of manifolds that can then be related to cognition and behavior. For example, Saggar et al. (2018) looked at topological structures in relation to cognitive performance fMRI data, finding that those with a more distributed topology showed better cognition (Figure 3). Additional features of estimated manifolds, such as switching, dwell time, and transitional probabilities, are important aspects that emphasize the temporal flows on the manifold. Along these lines, an emphasis on trial-by-trial time series, rather than simple averages of data, are preferable. Differences in average features may have some utility in selection of key nodes for network identification, but obliterate the higher order statistical moments of the data, which are central to SFM expression.

There is an additional aspect that highlights the uniqueness of the SFM framework, which is that the behavior that emerges from the brain must also be characterized as flows on manifolds. This enables a new level of analysis to better characterize brain-behavior relationship in terms how the specific evolution of flows on manifolds in brain constrain and are constrained by the flows on manifolds in behavior. Here there are fewer methods that map such interdependency between flows, though some candidates do exist (Breakspear & Terry, 2002; Flack, 2017; Terry & Breakspear, 2003).

This begs the question as to whether cognitive processes, such as memory and emotion can be characterized under the SFM framework. Although most behavioral measures of cognition are often single points, such as reaction time or accuracy of responses, the notion of mental flows is pervasive in theory (Spivey, 2007). A recent expression emphasizes a seamless flow capturing the process of moving between sensation and action and back where the lines between traditional states (e.g., sensation, perception, memory) is blurred if not absent. For cognitive processes this is a challenge, as they are not easily measured. However, their impact on ongoing behavior, such as eye movements or reaching (Song & Nakayama, 2009), has been used successfully to characterize the dynamics of processes and does give a potential access point for the creation of behavioral SFMs that can be linked to brain SFMs. The trajectories create a personal space that can be translated to a manifold. Across realizations, the flows along the manifold can then be related with the corresponding flow elicited in the brain—essentially mapping SFMs in behavior to those of the brain. This will yield new understanding of how the richness of behavior that we observe is enabled by the richness of brain dynamics that we measure.

## AUTHOR CONTRIBUTIONS

AR McIntosh: Conceptualization; Formal analysis; Methodology; Validation; Visualization; Writing – original draft; Writing – review & editing. Viktor Jirsa: Conceptualization; Formal analysis; Methodology; Validation; Visualization; Writing – original draft; Writing – review & editing.

## FUNDING INFORMATION

AR McIntosh, NSERC, Award ID: RGPIN-2018-04457. Viktor Jirsa, Horizon 2020 (http://dx.doi.org/10.13039/501100007601), Award ID: 720270. Viktor Jirsa, European Union’s Horizon 2020 Framework Programme for Research and Innovation, Award ID: 785907 (HBO SGA2).

## TECHNICAL TERMS

Refractory status epilepticus: Status epilepticus (a prolonged single seizure) that persists despite treatment.
Emergence: Behavior that results from nonadditive interactions of subordinate parts.
Motifs: Building blocks for systems that can be recombined in different ways to produce different outcomes.
Complex adaptive systems (CAS): Systems with a large number of components (agents) that adapt and learn. The behavior of the system cannot be easily distilled from focusing on each component.
